# COVID-19 mitigation strategies: A natural experiment highlighting the importance of structure in the prevention and treatment of childhood obesity

**DOI:** 10.1016/j.pmedr.2022.102023

**Published:** 2022-10-13

**Authors:** Keith Brazendale, Michael W. Beets, R. Glenn Weaver, Bridget Armstrong, Ethan T. Hunt

**Affiliations:** a Department of Health Sciences, College of Health Professions and Sciences, University of Central Florida, Orlando, FL, USA; b Department of Exercise Science, Arnold School of Public Health, University of South Carolina, Columbia, SC, USA; c Michael and Susan Dell Center for Healthy Living, University of Texas Health Science Center School of Public Health in Austin, Austin, TX, USA

**Keywords:** Childhood obesity, COVID-19, Weight gain, Structured days hypothesis

## Abstract

Societal restrictions due to the Coronavirus Disease 2019 (COVID-19), such as the closure of schools, childcare centers, and community programs, were implemented to prevent the spread of the disease and to protect the health and well-being of the population. These mitigation efforts drastically interrupted the day-to-day environments of children and adolescents and influenced how they spent the majority of their waking hours. Evidence shows on days when children and adolescents are in “structured” settings, such as school or other extracurricular programs or day camps, their obesogenic behaviors (i.e., sleep, physical activity, diet, and screen/media time) are more favorable than on days with less structure (e.g., summer days, weekends). Although obesity is driven by complex interactions between environmental, behavioral, biological, and genetic factors, COVID-19 pandemic closures emphasized the importance of daily structure. This short communication used a tertiary examination of the literature to show how societal restrictions and mitigation strategies resulted in significant increases in childhood and adolescent obesity on a global scale and highlights the importance of key underlying principles of the Structured Days Hypothesis (SDH). Closure of schools and other structured programs as a result of COVID-19 exposed youth to prolonged periods of less-structured environments as youth spent considerably more time at home than normal. Societal restrictions and mitigation strategies as a result of COVID-19 inadvertently demonstrated the importance of structure in shaping children’s health behaviors and weight-related outcomes. Public health practitioners and researchers should consider this framework in the development of interventions to prevent and treat obesity in youth.

## 1 Introduction

### 1.1 Background

The last two years presented an extreme disruption to the day-to-day environments of children and adolescents around the world due to Coronavirus Disease 2019 (COVID-19). Societal restrictions, such as the closure of schools, childcare centers, and community programs, were implemented to prevent the spread of the disease and to protect the health and well-being of the population. These mitigation efforts, while justified, disrupted children and adolescents’ typical day-to-day routines by the suspension of in-person school and attendance at other structured settings (e.g., daycare, community-based programs, extracurricular activities, and day camps). Global estimates report over 1.5 billion children from over 107 countries were impacted by national school closures in the early stages of the pandemic (circa March 2020) (Viner et al., 2020).

The importance of attending school, and other structured settings, in shaping daily routines and behaviors, and eventually health outcomes in children and adolescents (e.g., obesity) is well-documented in the literature (Brazendale et al., 2021) and longitudinal evidence from the past three decades shows children gain weight at a faster rate – hereon referred to as accelerated weight-gain – during periods of less-structured time (e.g., summer months when school is out) compared to when school is in session. (Lane et al., 2021) The *Structured Days Hypothesis* (SDH)^4^ posits the causal mechanism protecting children from accelerated weight-gain during school months (compared to summer vacation months) is the consistent presence of structure, routine, and/or regulation within each school day that influences obesogenic behaviors of children and adolescents (e.g., active/sedentary time, sleep, diet, screen/media time). (Brazendale et al., 2017) The onset of COVID-19 and the corresponding sweeping pandemic-related mitigation strategies can be viewed as one of the largest natural experiments of the SDH that tests how the removal of all structured settings outside the home impacts children and adolescents’ obesity-related behaviors and health outcomes. From a tertiary examination of the literature, the following short communication delineates how societal restrictions and mitigation strategies incorporated on a global scale highlight the key underlying principles of the SDH which resulted in a significant increase in the prevalence of childhood and adolescent obesity.

## 2 Childhood obesity and COVID-19: A natural experiment of the ‘Structured Days Hypothesis’

There is no question that the arrival of COVID-19 and the mitigation strategies (e.g., school closures, home quarantine) adopted on a global scale altered the daily routines for many children and adolescents. This was not without consequence. A tertiary examination of the literature uncovers a substantial number of studies that demonstrate significant accelerated weight-gain and an increase in the proportion of children and adolescents classified as overweight or obese (Woolford et al., 2021, Chang et al., 2021) compared to the previous two years during COVID-19. Further, numerous studies published data (e.g., cross-sectional, longitudinal) or synthesized best-available evidence (e.g., narrative, scoping, systematic reviews) highlighting unfavorable changes in children and adolescents’ obesogenic behaviors during pandemic months. (Okely et al., 2021, Viner et al., 2021, Kharel et al., 2022) Some key findings show that, during the pandemic, the greatest increases in weight occurred in younger children (5 to 11 years old). (Woolford et al., 2021) Additionally, children of parents reporting higher levels of stress or less access to outdoor activity space were less likely to meet World Health Organization global guidelines for physical activity, sedentary screen time, and sleep. (Okely et al., 2021).

*As a scientific community, should we not have anticipated these disturbing health outcomes?* A common denominator faced by many children and adolescents from all over the world was the unexpected and widespread interruption to their ‘typical’ day-to-day structure and routines, as schools and school-like environments closed for public access. The role consistent access to ‘structure’ can have in regulating an individual’s behavior on a daily basis (i.e., a structured day (Brazendale et al., 2017) and the continuing impact it can have on health outcomes has never been more evident. This central tenet of the SDH, for the most part, anticipated this.

An argument can be made that the removal of access to structured settings due to the COVID-19 pandemic is analogous to what the scientific community has observed when children are removed from the consistent access to school – and other school-related programs and settings – for extended periods of time, such as summer break. (Rundle et al., 2020) In its simplest form, the SDH suggests attending structured settings provide an underlying structure to a child’s day, and, therefore, mitigates the occurrence of unhealthy behaviors such as prolonged sitting and screen time, reduced health-enhancing physical activity, irregular sleep patterns, and less-healthful and/or irregular diets. These hallmark unhealthy behaviors, which are observed more frequently during weekends and summer vacation, (Brazendale et al., 2021, Olds et al., 2019) occur at an increasing rate during normal at-home conditions (i.e., before COVID-19). It’s of little surprise during pandemic months, when restrictions were enforced with schools and other structured settings closed indefinitely, the confinement of children and adolescents to a less-structured environment in the home for prolonged periods of time resulted in similar negative health effects.

According to the SDH, the typical weekend and summer day environment allows for more discretionary time and with this, freedom, or variation around when children and adolescents wake-up in the morning. This can impact other behaviors that occur throughout the day (e.g., displaces physical activity, shifts in meal timing). During the waking hours of a summer day children have greater autonomy (more open-endedness) regarding how they spend their time and may choose more sedentary pursuits. In contrast, on a school day, physical activity is either intentionally (e.g., recess, physical education) or unintentionally (e.g., active commuting/transitions at school) promoted. During less-structured days, consistency around meal timing and meal access are compromised and the likelihood of sedentary pursuits occurring and displacing physical activity are much higher. Sedentary pursuits, such as screen/media time, may involve other co-occurring behaviors like excess snacking on calorie-dense foods. Lastly, less-structured days often result in later bedtimes, displacing crucial sleep time with yet more sedentary screen/media time activities (Brazendale et al., 2017).

Evidence shows children and adolescents’ physical activity levels decreased, (Viner et al., 2021, Kharel et al., 2022, Burkart et al., 2022) sedentary time increased, (Okely et al., 2021, Burkart et al., 2022) and screen/media time increased (Neshteruk et al., 2021). In addition, sleep duration and timing was longer and bed/wake times later, (Okely et al., 2021, Burkart et al., 2022) dietary habits were altered (Burkart et al., 2022, Jansen et al., 2021), and access to consistent school-based meals for children and adolescents from low-income households diminished, increasing food insecurity during the pandemic (Adams et al., 2021). Physical activity was greatly impacted as children couldn’t attend schools, sport-related programs/activities, play with their friends, or use local parks. Children accrue most of their sedentary time and sleep at home and the home quarantine/lockdown rules increased this substantially (Okely et al., 2021, Burkart et al., 2022, Neshteruk et al., 2021). Longer than normal amounts of time spent at home during lockdown exposed them to higher amounts of potentially unsupervised screen/media time, combined with a shift to ‘virtual schooling’, further promoting increased sedentary time (Okely et al., 2021). Without an imposed school start time, children and adolescents sleep timing (bed/wake times) shifted later, while sleep duration increased (Okely et al., 2021, Burkart et al., 2022). Longer duration of sleep is not particularly surprising, as a shift in sleep timing can allow for a more natural, biological, sleep-wake cycle to occur. The removal of having to attend a school or program in the morning was not without consequence. Studies show later bedtimes and later sleep onset, independent of sleep duration or waketime, is associated with less physical activity, greater caloric intake, and increased screen/media time in obese adolescents (Adamo et al., 2013). Certain countries enacted stimulus bills and government assistance programs to provide economic relief and to address food insecurity, albeit later into the pandemic, yet the closure of schools ultimately reduced access to free or subsidized meals for millions of children, shifting the reliance of daily meals/nutrition to the home environment and increasing food insecurity during COVID-19 (Rundle et al., 2020, Adams et al., 2021). This paralleled an increase in households snacking on ultra-processed, calorie-dense, shelf-stable comfort foods, which resulted in a negative impact on diet quality for children and adolescents (Burkart et al., 2022, Jansen et al., 2021).

Given the length of the time children and adolescents were removed from consistent structure to their day, one could argue the impact was intensified, and the health consequences much greater, in comparison to what we observe over summer. In essence, families were tasked with managing and emulating everything that encompasses a ‘school day’, but in their homes, (Bates et al., 2020) whilst simultaneously trying to navigate the stress and anxiety of living through a pandemic, and the major disruption to their own ‘typical schedules’. Fundamentally, an extreme and abrupt change in the consistency of an environment any individual is exposed to on a daily basis will cascade to alterations in behaviors that impact and misalign physiological processes in the body (i.e., disruption to the circadian clock) that play a key role in the expression of obesity (Moreno et al., 2019). School closures as a result of COVID-19 contributed toward a shift in bed- and wake-times, and less consistency with meal and/or snack timing and available physical activity opportunities (Okely et al., 2021, Burkart et al., 2022, Neshteruk et al., 2021, Jansen et al., 2021, Bates et al., 2020). Variability in the timing of meals, sleep, and activity patterns results in a misalignment between the body’s central and peripheral clocks that can lead to negative health outcomes, such as obesity (Moreno et al., 2019). Whereas during ‘typical’ school months the days could be considered more scheduled and consistent, largely determined by children having to attend school and engage in all of the elements that come before (morning routine) and after (evening routine) attending.^4^.

**Fig. 1:**
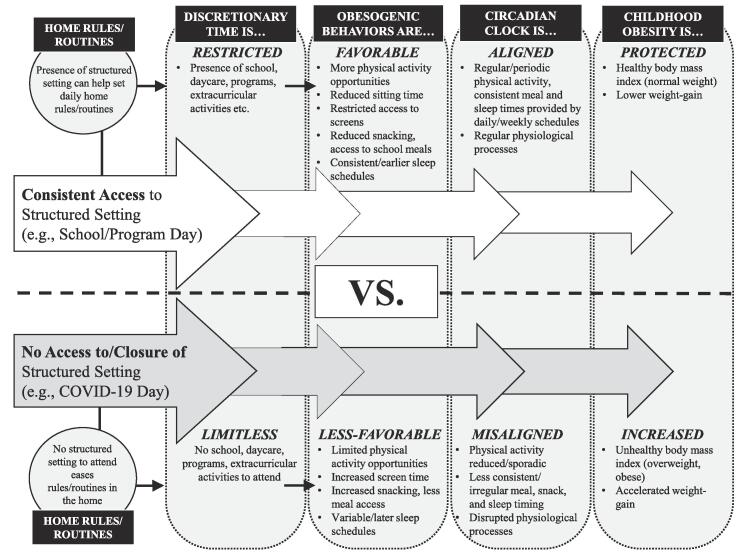
Conceptual model illustrating the impact of COVID-19-related school and community-based program closures on childhood obesity in comparison to a typical structured day (e.g., school/program day).

Emerging evidence indicates the presence of structured settings may also influence the amount of structure that occurs in the family home (defined as the rules, routines, and household order). Studies have explored the role of setting rules, and the level of household/family routines and functioning, as determinants or correlates of obesity in children and adolescents (Anderson and Whitaker, 2010). During COVID-19, families experienced strain and disruption to their family system, felt out of control, and reported a range of variation in how they managed scheduling demands (or a lack thereof) and their approach to parenting and routines (Larson et al., 2021). Conversely, children and adolescents from households that managed to maintain a relatively ‘normal’ level of order and consistency whilst implementing some ‘school-like’ daily schedule throughout the pandemic – a strategy suggested by many experts in the field – may have manifested in more favorable health outcomes for children or adolescents. Unfortunately, studies presenting such data are absent from the literature. Fig. 1 illustrates a conceptual model of the impact of COVID-19-related school and community-based program closures on aspects of the home environment, obesogenic behaviors of children, the body’s circadian clock, and ultimately childhood obesity, in comparison to times when children have more consistent access to structured settings (i.e., pre-COVID-19). It must be noted that other mechanisms, beyond the SDH, could explain the rapid increase in children and adolescents’ weight-status during the pandemic (e.g., COVID-19 stress-induced induces neuroimmunoendocrine changes), however, the evidence presented herein, makes an argument for the influential role of structure, as defined by the SDH.

## 3 Implications and conclusions

The COVID-19 pandemic highlighted the importance of consistent daily structure and routines – core components of the SDH – in the development of obesity in children and adolescents across the world. Whilst the SDH does not completely explain the accelerated weight-gain observed during COVID-19, an argument can be made that it was one of the key driving frameworks. The pandemic reinforced the inherent ‘healthy’ and positive impact a structured day provides that influences obesogenic behaviors and physiological processes in the human body related to health outcomes in children and adolescents. We recognize the day-to-day components and segments of structured days will differ, nonetheless, it is the mere presence of having to attend a structured setting that appears to regulate the occurrence of unhealthy behaviors.

In summary, societal restrictions and mitigation strategies have impacted child and adolescent obesity on a global scale. The SDH has garnered increasing interest within the scientific community during the pandemic as researchers have studied the indirect impact of COVID-19 on children’s health. Explicitly, the COVID-19 pandemic demonstrated the importance of structure in shaping children’s health behaviors and weight-related outcomes. Public health practitioners and researchers should consider this framework in the development of interventions to prevent and treat obesity in youth. Opportunities for further research exist, such as how COVID-19 has impacted families with children and adolescents from low-income countries, longitudinal studies that examine the lasting impact of the pandemic years on weight-related outcomes and behaviors, and the relationship between physical and mental health during extended periods of less-structured time.

## 4 Availability of data and materials

Data sharing is not applicable to this article as no datasets were generated or analyzed during the current study.

## Declaration of Competing Interest

The authors declare that they have no known competing financial interests or personal relationships that could have appeared to influence the work reported in this paper.

## Data Availability

No data was used for the research described in the article.
